# Trajectories and Determinants of Posttraumatic Stress in Disaster Survivors: A Group-Based Trajectory Modeling Study

**DOI:** 10.1097/jnr.0000000000000762

**Published:** 2026-09-04

**Authors:** Ahram IM, Chin Kang KOH

**Affiliations:** 1 College of Nursing & The Research Institute of Nursing Science, Seoul National University, Seoul, Republic of Korea

**Keywords:** disaster survivor, posttraumatic stress, group-based trajectory modeling

## Abstract

**Background:**

Disasters can result in long-term posttraumatic stress (PTS), the trajectory of which may vary widely among individuals. Although typical patterns of PTS have been suggested in prior studies, scholarly consensus on pattern classification and related factors remains limited.

**Purpose:**

This study was developed to identify the general patterns of PTS in disaster survivors over time using trajectory modeling and to examine the characteristics of each trajectory group.

**Methods:**

A secondary analysis was conducted on original data from the Long-term Survey on the Change of Life of Disaster Victims implemented by the National Disaster Management Research Institute of South Korea. PTS trajectories with similar trends were grouped together using Group-Based Trajectory Modeling (GBTM), and the characteristics of each trajectory group were identified using multinomial logistic regression analysis.

**Results:**

The GBTM analysis identified four distinct PTS trajectory groups. Group 1, which captured the largest number of individuals, showed stable, low-level PTS over time (Group 1: resistance 66.1%, *n* = 698); Group 2 captured individuals with rising PTS trajectories over time (Group 2: delayed, 11.1%, *n* = 101); Group 3 captured individuals with an initially high level of PTS and a trajectory of recovery over time (Group 3: recovery, 16.4%, *n* = 161); and Group 4, representing the smallest number of individuals, showed stable, high-level PTS over time (Group 4: chronic, 6.4%, *n* = 65). The adjusted analysis showed that Groups 2–4 differed from Group 1 in several variables, including resilience and disaster-related harm variables.

**Conclusion/Implications for Practice:**

The representative trajectories of PTS in disaster survivors and their associated characteristics were identified in this study. The findings indicate individuals with initially low levels of PTS may experience rising symptom severity (Group 2) and that those with initially high levels of PTS may face slower-than-expected recoveries (Group 4). Based on the findings, tailored interventions accounting for the group-specific risk factors identified in this study are needed.

## Introduction

Disasters often lead to long-term mental health issues, notably posttraumatic stress disorder (PTSD), that significantly impair social functioning in survivors (Norris et al., 2002; Saeed & Gargano, 2022). PTSD, typically diagnosed through self-report questionnaires and clinical interviews, is generally classified using cutoff scores. However, the accuracy of single-time assessments is limited, as posttraumatic stress (PTS) fluctuates dynamically over time (First, 2024; North & Oliver, 2013), complicating the implementation of effective early-intervention measures. This issue has prompted researchers to track PTS trends over time (Norris et al., 2009), facilitating the development of tailored support strategies (Lai et al., 2021).

Although PTS generally declines over time, some individuals experience prolonged or worsening symptoms (Neria et al., 2008; Sakuma et al., 2020). The existence of delayed PTS trajectories remains debated, with some studies supporting the pattern (Joshi & Cerdá, 2017; Heir et al., 2021) and others not (Lai et al., 2021; Sakuma et al., 2020). While four trajectory classification groups (resistance, recovery, delayed, chronic) are most commonly cited (Bryant, 2021), up to seven have been identified in some studies (Norris et al., 2009; Sakuma et al., 2020).

Disaster research faces methodological challenges that contribute to inconsistencies in findings and conclusions (North et al., 2012). The variation in PTS trajectories reported in the literature may stem from a focus on a single disaster incident or from limited longitudinal follow-up (First, 2024; Joshi & Cerdá, 2017). Also, reliable predictors of PTS trajectories have been poorly corroborated. Although frequently cited predictors include female sex, older age, lower economic status, and severe disaster exposure (Fatema et al., 2021; First, 2024; Tahan et al., 2021), the significance of broader domains such as trauma characteristics, individual predispositions, and societal context remains unclear (North et al., 2012; Tahan et al., 2021). Although age-related variation in stress response due to stage-of-life characteristics such as physical and cognitive functions has also been suggested, the extant evidence remains inconclusive (Lai et al., 2021).

Considering the complex interplay of individual, societal, and environmental factors, comprehensive approaches are important in disaster research (Neria et al., 2008). This study was developed to address these gaps by conducting a secondary analysis of multiyear data from survivors of typhoons, heavy rains, fires, and earthquakes in South Korea. Each participant experienced a single disaster type, and the sample expanded over four survey waves. The data set includes conventional PTS-related variables such as demographics, disaster experiences, psychological health, and socioeconomic factors as well as potentially relevant variables to capture a more diverse and comprehensive perspective. For example, media satisfaction was included as a variable because inadequate disaster coverage may potentially lead to the secondary victimization of survivors and bereaved families (Park et al., 2019). The aim of this study was to identify PTS trajectory patterns and examine group characteristics to inform effective intervention strategies.

## Methods

### Study Design and Sample Size

A secondary analysis was conducted on data from the Long-term Survey on the Change of Life of Disaster Victims, carried out annually from 2016 to 2019 in South Korea by the National Disaster Management Research Institute. For this survey, a multiple-panel design was used to provide a broad perspective on disaster survivors and included household members aged 13 and older who had received legal compensation for disasters occurring between 2012 and 2017. The survey sample was stratified by sex, age, and region for proportionality.

The survey data set exhibits both panel and trend study characteristics, and to prevent data reduction and ensure a wider range of subjects, the sample size varied across each wave. Disaster timing ranged from zero to five years post event (average years after disaster: first survey wave = 2.93, second = 2.41, third = 3.33, fourth = 4.41). A statistical expert was consulted to control these variations. Of the 3,288 participants in the survey, only those with complete PTS data across at least three waves were included in this study, yielding a final sample of 1,025.

### Instruments

PTS was measured in this study using the Korean version of the Impact of Event Scale-Revised (IES-R-K; Eun et al., 2005), a 22-item self-report scale with a potential total score range of 0–88. Total scale scores above 17 were interpreted as indicating PTSD risk and over 25 indicating probable PTSD. The Cronbach’s alpha for the IES-R-K ranged from .69 to .83. Other study variables, including resilience (measured by the Average score of the Brief Resilience Scale) and social adjustment (measured by the Average score of the Work and Social Adjustment Scale) are described in detail in Supplemental Digital Content 1 (http://links.lww.com/JNR/A15).

### Data Analysis

Data were analyzed using R 4.2.3 (R Foundation for Statistical Computing, Vienna, Austria) and Stata v18.0 (StataCorp LLC, College Station, TX, USA). A two-tailed significance level of *p* < .05 was used. PTS trajectories were examined using Group-Based Trajectory Modeling (GBTM) in accordance with Nagin’s two-step model selection procedure of first estimating the number of groups and then determining polynomial orders for each group (Nagin, 2005). The optimal model was selected based on the Bayesian Information Criterion (BIC), a relatively conservative and popular GBTM criterion (Nagin, 2005). Also, all classified groups were required to comprise at least 5% of the total sample (Nagin, 2005). Model validity was assessed using Average Posterior Probability (threshold ≥0.7) and Odds of Correct Classification (high accuracy >5.0).

Multinomial logistic regression was used to examine the factors associated with trajectory group membership. The low-risk PTS group (Group 1) was set as the reference, and group membership was the categorical outcome variable. Sex, age, economic status, and time elapsed since disaster incident were included as prespecified covariates based on prior literature and statistical consultation. Age was categorized following the practice in previous studies into adolescent, adult, and older adult subgroups to reflect life-stage differences. Economic status was treated as a continuous monthly household income variable.

### Ethical Considerations

This study was approved by the institutional review board of the authors’ affiliated university (IRB No. E2401/002-003). Usage of the survey data was authorized by the National Disaster Management Research Institute, which conducted the original survey. All data were anonymized before analysis.

## Results

### Results of the Group-Based Trajectory Model

**Figure 1:**
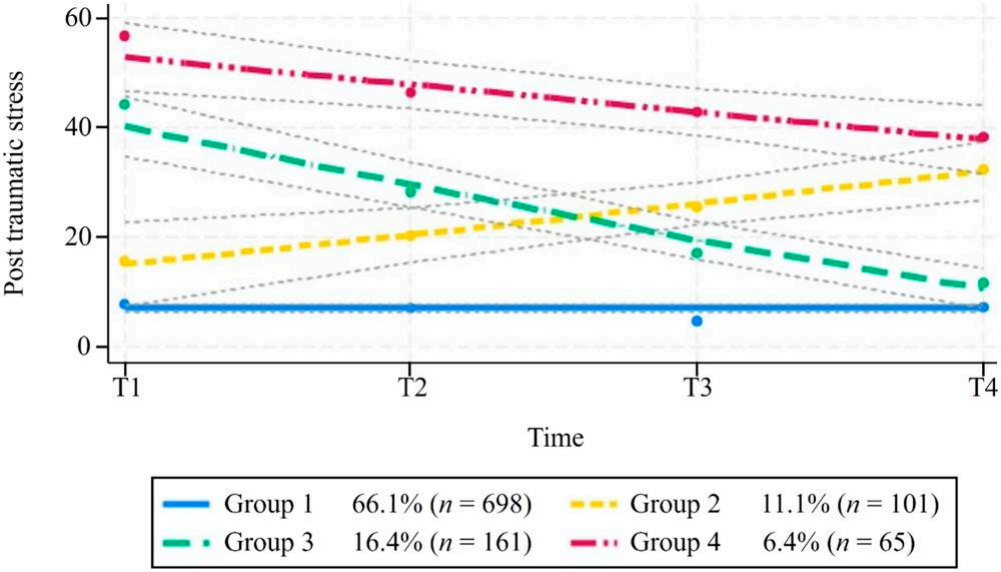
Trajectory Plot of the Four Groups Identified in the Study Data Note. Group 1 = resistance (range: 4.8-7.9); Group 2 = delayed (range: 15.8-32.2); Group 3 = recovery (range: 11.6-44); Group 4 = chronic (range: 48.1-56.8); T1 = first survey wave; T2 = second survey wave; T3 = third survey wave, T4 = fourth survey wave; Points = observed value; Line = predicted value; Gray area = confidence interval.

Four trajectories were identified with the polynomial orders of 0, 1, 1, and 2. This four-group solution had the smallest absolute BIC value while meeting the minimum sample size requirement for all groups. Model fit indices are provided in Table 1 and the group trajectories are shown in Figure 1. The trajectories included: stable low PTS (Group 1, resistance: 66.1%), increasing PTS (Group 2, delayed: 11.1%), decreasing PTS (Group 3, recovery: 16.4%), and persistently high PTS (Group 4, chronic: 6.4%).

**Table 1:** Fit indices for the Study Model by Group Number and Shape

No. Groups	Trajectory Shapes ^a^	BIC ^b^ (*N* = 3,554)	BIC ^c^ (*N* = 1,025)	AIC	Log Likelihood
1	0	−12,231.38	−12,230.14	−12,225.21	−12,223.21
2	0 2	−11,940.56	−11,936.83	−11,922.03	−11,916.03
3	0 2 2	−11,924.34	−11,918.12	−11,893.46	−11,883.46
**4**	**0 2 2 2**	**−11,909.91**	**−11,901.21**	**−11,866.68**	**−11,852.68**
5	0 2 2 2 2	−11,941.66	−11,930.47	−11,886.08	−11,868.08
6	0 2 2 2 2 2	−11,920.76	−11,907.08	−11,852.83	−11,830.83
7	0 2 2 2 2 2 2	−11,929.54	−11,913.38	−11,849.26	−11,823.26

Results for the four groups					
4	0 2 2 2	−11,909.91	−11,901.21	−11,866.68	−11,852.68
	0 0 0 0	−11,946.45	−11,941.48	−11,921.75	−11,913.75
	0 0 0 1	−11,950.54	−11,944.95	−11,922.75	−11,913.75
	0 0 0 2	−11,934.42	−11,928.21	−11,903.54	−11,893.54
	0 0 1 2	−11,925.92	−11,919.08	−11,891.95	−11,880.95
	**0 1 1 2**	**−11,901.71**	**−11,894.25**	**−11,864.65**	**−11,852.65**
	0 1 1 1	−11,928.41	−11,921.57	−11,894.44	−11,883.44
	0 0 2 2	−11,909.67	−11,902.21	−11,872.62	−11,860.62
	0 1 2 2	−11,913.74	−11,905.66	−11,873.60	−11,860.60

	**Group 1**	**Group 2**	**Group 3**	**Group 4**	
AvePP	0.925	0.715	0.730	0.784	
OCC	5.776	22.916	14.492	53.482	

*Note.* BIC = Bayesian information criterion; AIC = Akaike information criterion; AvePP = average posterior probability; OCC = odds of correct classification. Boldface indicates the final selected model based on model-fit indices and model-selection criteria. ^a^ 0 = zero order, 1 = linear, 2 = quadratic. ^b^ for the total number of data points. ^c^ for the total number of subjects.

### Characteristics of the Four Trajectory Groups

Group 1 represented the most favorable outcome for disaster survivors. Thus, multinomial logistic regression was conducted using PTS trajectory group membership as the outcome variable, with Group 1 set as the reference category. In the adjusted analysis, significant differences in six of the study variables, including economic status, resilience, time elapsed since disaster incident, personal injuries or illness, perceived damage, and media satisfaction, were observed between each of the three groups (Groups 2–4) and Group 1. The other study variables were either similar or differed only in specific group comparisons, with the results detailed in Table 2.

**Table 2:** Results of Multinomial Logistic Regression for Group Characteristics

			Unadjusted Results			Adjusted Results		
Variable (Reference)	Category	Group ^a^	*OR*	95% CI	*p*	*OR*	95% CI	*p*
Sex (Men)	Female	2	1.56	[1.019, 2.387]	**.040**	1.53	[0.991, 2.356]	.055
		3	1.20	[0.853, 1.696]	.292	1.21	[0.847, 1.716]	.299
		4	2.89	[1.629, 5.124]	**.000**	2.78	[1.548, 5.003]	**.000**
Age		2	1.03	[1.017, 1.046]	**.000**	1.03	[1.008, 1.059]	**.010**
		3	1.01	[0.999, 1.019]	.075	1.01	[0.994, 1.029]	.184
		4	1.03	[1.012, 1.047]	**.001**	1.04	[1.012, 1.074]	**.006**
Living alone (no)	Yes	2	2.32	[1.135, 4.721]	**.021**	1.23	[0.575, 2.635]	.592
		3	2.38	[1.313, 4.328]	**.004**	2.01	[1.051, 3.834]	**.035**
		4	4.74	[2.361, 9.497]	**.000**	2.62	[1.186, 5.795]	**.017**
On social welfare (no)	Yes	2	5.92	[2.277, 15.379]	**.000**	4.14	[1.514, 11.291]	**.006**
		3	1.31	[0.356, 4.802]	.687	1.06	[0.279, 4.039]	.931
		4	4.51	[1.374, 14.806]	**.013**	3.02	[0.852, 10.665]	.087
Economic status		2	0.72	[0.620, 0.842]	**.000**	0.72	[0.612, 0.856]	**.000**
		3	0.91	[0.811, 1.009]	.072	0.86	[0.755, 0.969]	**.014**
		4	0.69	[0.573, 0.840]	**.000**	0.64	[0.515, 0.795]	**.000**
History of mental illness (no)	Yes	2	3.33	[1.236, 8.962]	**.017**	1.95	[0.700, 5.436]	.201
		3	2.76	[1.122, 6.760]	**.027**	1.73	[0.691, 4.345]	.241
		4	4.39	[1.514, 12.730]	**.006**	1.92	[0.629, 5.865]	.252
Resilience		2	0.36	[0.254, 0.513]	**.000**	0.42	[0.292, 0.616]	**.000**
		3	0.33	[0.241, 0.437]	**.000**	0.33	[0.242, 0.456]	**.000**
		4	0.13	[0.083, 0.201]	**.000**	0.15	[0.093, 0.239]	**.000**
Time elapsed since disaster incident		2	0.81	[0.700, 0.931]	**.003**	0.79	[0.686, 0.919]	**.002**
		3	0.69	[0.611, 0.788]	**.000**	0.68	[0.599, 0.776]	**.000**
		4	0.60	[0.485, 0.742]	**.000**	0.58	[0.470, 0.718]	**.000**
Life threatened nno)	Yes	2	1.45	[0.931, 2.257]	.101	1.42	[0.900, 2.230]	.083
		3	2.38	[1.675, 3.387]	**.000**	2.27	[1.578, 3.251]	**.000**
		4	3.00	[1.791, 5.013]	**.000**	2.93	[1.711, 5.008]	**.000**
Witnessed injury or death of acquaintance (no)	Yes	2	0.66	[0.232, 1.885]	.439	0.76	[0.262, 2.190]	.608
		3	2.95	[1.733, 5.008]	**.000**	3.10	[1.779, 5.371]	**.000**
		4	2.91	[1.384, 6.129]	**.005**	4.00	[1.795, 8.891]	**.001**
Personal injury or illness (no)	Yes	2	3.15	[1.740, 5.692]	**.000**	3.06	[1.667, 5.624]	**.000**
		3	4.64	[2.898, 7.426]	**.000**	4.74	[2.904, 7.729]	**.000**
		4	7.42	[4.092, 13.477]	**.000**	7.33	[3.877, 13.846]	**.000**
Family member's injury or illness (no)	Yes	2	0.52	[0.122, 2.232]	.381	0.69	[0.157, 2.992]	.615
		3	2.85	[1.492, 5.452]	**.002**	3.50	[1.761, 6.973]	**.000**
		4	5.27	[2.469, 11.235]	**.000**	8.60	[3.655, 20.247]	**.000**
Extent of financial loss		2	1.01	[0.999, 1.013]	.007	1.01	[1.000, 1.013]	.056
		3	1.01	[1.007, 1.017]	**.000**	1.01	[1.007, 1.017]	**.000**
		4	1.02	[1.010, 1.021]	**.000**	1.02	[1.010, 1.021]	**.000**
Perceived damage		2	1.32	[1.059, 1.637]	**.000**	1.34	[1.069, 1.669]	**.011**
		3	1.61	[1.330, 1.956]	**.013**	1.63	[1.339, 1.982]	**.000**
		4	1.93	[1.413, 2.627]	**.000**	1.96	[1.426, 2.691]	**.000**
Used temporary housing (no)	Yes	2	5.43	[2.188, 13.450]	**.000**	1.94	[0.970, 3.896]	.061
		3	3.01	[1.432, 6.347]	**.000**	2.79	[1.636, 4.754]	**.000**
		4	2.51	[1.660, 3.773]	**.000**	4.35	[2.162, 8.758]	**.000**
Relocation of residence (no)	Yes	2	0.91	[0.455, 1.829]	.797	0.83	[0.408, 1.974]	.612
		3	3.53	[2.333, 5.339]	**.000**	2.82	[1.833, 4.341]	**.000**
		4	2.94	[1.610, 5.376]	**.000**	2.33	[1.229, 4.433]	**.010**
Separation from family (no)	Yes	2	1.86	[0.679, 5.099]	.227	2.17	[0.617, 4.993]	.292
		3	3.40	[1.669, 6.945]	**.001**	3.59	[1.338, 6.032]	**.007**
		4	4.31	[1.742, 10.687]	**.002**	4.75	[1.189, 8.449]	**.021**
Social adjustment		2	0.78	[0.611, 0.992]	**.043**	0.86	[0.666, 1.114]	.256
		3	0.45	[0.380, 0.542]	**.000**	0.43	[0.357, 0.523]	**.000**
		4	0.26	[0.203, 0.337]	**.000**	0.25	[0.192, 0.334]	**.000**
Social support		2	0.87	[0.665, 1.143]	.322	0.96	[0.719, 1.275]	.767
		3	0.78	[0.626, 0.970]	**.026**	0.78	[0.617, 0.986]	**.038**
		4	0.68	[0.493, 0.926]	**.015**	0.70	[0.490, 1.002]	.051
Community resilience		2	0.57	[0.416, 0.773]	**.000**	0.58	[0.418, 0.810]	**.001**
		3	0.75	[0.581, 0.966]	**.026**	0.88	[0.672, 1.148]	.342
		4	0.39	[0.264, 0.565]	**.000**	0.45	[0.295, 0.670]	**.000**
Media satisfaction		2	0.44	[0.316, 0.614]	**.000**	0.47	[0.309, 0.616]	**.000**
		3	0.55	[0.415, 0.721]	**.000**	0.57	[0.427, 0.756]	**.000**
		4	0.37	[0.248, 0.547]	**.000**	0.36	[0.231, 0.551]	**.000**

*Note. ORs* indicates the change in odds of belonging to Group k versus Group 1: per one-unit increase for continuous variables and relative to the reference category for categorical variables. *OR* = odds ratio. Covariates in the adjusted analysis: sex, age, economic status, and time elapsed since disaster incident, Group 1 = resistance, Group 2 = delayed, Group 3 = recovery, Group 4 = chronic.

^a^ Group 1 = reference group.

## Discussion

The PTS trajectories of disaster survivors in this study were classified into four distinct groups, which aligns with the prototypical patterns described in prior research (Bryant, 2021). As noted in the Results section, several variables, including economic status and resilience, significantly distinguished all trajectory groups from the low-risk Group 1, underscoring the multidimensional influences on PTS outcomes. Thus, individuals with these characteristics may be at higher risk of PTS, even though predicting specific trajectories may require additional variables. Beyond these shared determinants, group-specific differences were also observed and are discussed below.

### Group 1: Resistance Group

The majority of the disaster survivors (66.1%) showed stably low PTS, which is consistent with studies reporting minimal PTS in two-thirds of disaster survivors (Tahan et al., 2021). This reinforces the idea that, while disasters impact large populations, targeted psychological support should focus on at-risk subgroups rather than on all survivors.

Group 1 demonstrated favorable characteristics, including higher resilience, better economic status, and less disaster-related harm (injuries, perceived damage) than the other groups. This finding aligns with prior evidence pointing to resilience as a key factor in ameliorating PTS (First, 2024; Saeed & Gargano, 2022). The relationships identified in this study between PTS and economic status as well as between PTS and the extent of disaster harm also align with the findings of previous studies (First, 2024; Lee et al., 2022). Meanwhile, media satisfaction, a less-explored factor, differed significantly in the three comparison groups from Group 1. Inaccurate or intrusive media coverage may heighten stress, whereas timely and accurate reporting may foster a sense of community among survivors. Further research is needed to clarify the influence of this variable on PTS.

### Group 2: Delayed Group

Findings regarding this group, which exhibits delayed PTS, vary across studies, with some confirming its existence (Bonde et al., 2022; Heir et al., 2021) and others casting doubt on it (Johannesson et al., 2015; Lai et al., 2021; Sakuma et al., 2020). Given that inconsistencies may result from differences in methodology, follow-up periods, and study populations, this long-term, multidisaster study likely captured cases that shorter studies may miss.

The mechanism underlying delayed PTS remains debated, with one hypothesis suggesting that individuals who initially adapt well may later develop symptoms due to subsequent stressors (Johannesson et al., 2015), potentially due to renewed traumatic exposure, especially when these new stressors remind the individual of their prior disaster experience (Bonde et al., 2022). As the data set used in this study did not include details on follow-up events, these details may be included in future research to consider related mechanisms to further deepen understanding.

The significant risk factors for inclusion in Group 2 were: older age, low resilience, poor economic status, and receiving social welfare. As noted in a previous study, less-severe initial exposure is a common feature of delayed onset (Bonde et al., 2022). Also, disaster-related hardships, except for personal injury and perceived damage, were not identified as statistically significant predictors for Group 2. Despite its relatively small proportion (11.1%) of the data set, Group 2 is at high risk of being overlooked in early post-disaster screenings as they exhibit low initial PTS levels (similar to Group 1). Therefore, follow-up monitoring is necessary for individuals with low PTS, especially for those with Group 2 profile characteristics.

### Group 3: Recovery Group and Group 4: Chronic Group

Both of these groups experience high initial PTS, with Group 3 only experiencing gradual recovery. Group 4, while representing the smallest proportion of survivors (6.4%), requires the most intensive, long-term care. Therefore, screening survivors for Group 4 predictors is important to implementing effective, targeted intervention strategies. Groups 3 and 4 share significant disaster-related characteristics, including high perceived threat to life, witnessing casualties, and financial loss. However, the magnitude of the associations was generally larger for Group 4 than for Group 3 for several risk factors, although direct comparisons between these groups were not conducted.

Groups 3 and 4 also reported significantly higher family and housing-related problems such as having injured family members, being separated from family, and experiencing relocation or temporary housing, which are considered to be among the most distressing consequences of disasters (Neria et al., 2008). Neurobiological evidence suggests highly distressing experiences may imprint on the amygdala, prolonging the duration of perceived distress (Raise-Abdullahi et al., 2023). Individuals exposed to severe trauma may not only start with higher PTS but also recover more slowly, necessitating proactive long-term interventions, including behavioral therapy and pharmacological support. Although predisaster psychiatric disorders did not differ significantly among the groups in this study, related findings should be interpreted cautiously as the data only reflect disorders experienced within the three-month period before the disaster. The results of prior research suggest broader psychiatric history may be a significant predictor of PTS (North et al., 2012; Saeed & Gargano, 2022).

Furthermore, the findings of this study confirm the important influence of social factors. Both Group 3 and 4 reported lower levels of social adaptation, with Group 3 reporting less social support and Group 4 exhibiting weaker community resilience. Consistent with studies showing strong social networks mitigate PTS symptoms (Lee et al., 2022), the findings of this study emphasize the need for interventions targeting social integration and support. However, further research is needed to determine why these variables were significant in only one group and whether they distinguish Groups 3 and 4. Finally, several demographic variables were identified that may be significant in distinguishing individuals between Groups 3 and 4. Living alone was common in both Group 3 and Group 4, suggesting social isolation may contribute to higher PTS levels. However, unlike Group 3, Group 4 included significantly higher percentages of women and older-age individuals, which aligns with previous studies (Heir et al., 2021; Norris et al., 2002). Biological mechanisms are often invoked to explain prolonged distress, and females may have greater neurobiological sensitivity to trauma due to the stronger activation of their amygdala (Raise-Abdullahi et al., 2023), while age-related hippocampal volume deficits may impair stress regulation in older adults (Woon et al., 2010). Therefore, women and older adults with higher perceived levels of PTS may experience slower recovery. Further research is necessary to determine the specific age threshold of this effect.

### Strengths and Limitations

In this study, secondary data from typhoon, heavy rain, fire, and earthquake disasters were analyzed. Thus, the generalizability of findings to survivors of other disaster types may be limited. Also, the multiyear nature of the survey and variability in participants and variables constrained panel consistency. In addition, the variability in time elapsed between disasters and survey responses potentially affected PTS trends despite the related control measures used. Lastly, reliance on self-reported data, including damage, economic status, and medical history, may have biased the data. Future research should incorporate objective data and more consistent longitudinal tracking of diverse disasters.

### Implications for Practice

This study provides valuable insights for nursing practice and disaster management by identifying PTS trajectories and their characteristics. Understanding these patterns can enable the implementation of a phased approach to mental health intervention.

In the predisaster stage, identifying risk factors for delayed and chronic PTS can help tailor preparedness programs, especially for vulnerable groups such as females, the elderly, and low-income populations. During the response stage, early screening can help differentiate high-risk individuals from resilient ones, guiding the delivery of targeted psychological first aid and crisis counseling. In the post-disaster recovery stage, long-term monitoring is crucial to detecting delayed PTS, while collaboration with social services and emergency management teams is essential to addressing the multifaceted needs of those experiencing chronic PTS.

### Conclusions

In this study, four distinct PTS trajectories were identified, offering actionable insights into long-term post-disaster mental health patterns. The findings underscore the importance of implementing interventions tailored to the specific characteristics of each trajectory group and highlight the variables warranting further targeted study and clarification.

## Supplementary Material
