# Supplementary material for: Trajectories and Determinants of Posttraumatic Stress in Disaster Survivors: A Group-Based Trajectory Modeling Study
Source: J Nurs Res. 2026 Sep 4;34(5):e482. doi: 10.1097/jnr.0000000000000762 (PMC13608321; doi:10.1097/jnr.0000000000000762)
Supplement: Supplementary file 1 [file jnr-34-e482-s001.docx]

**Appendix**

*Complete List of Scale Items*

Item

Factor 1: Comprehension and evaluation of general information

1. Understanding the content of HPV prevention information from different sources is…

2. Understanding the routes of transmission for HPV infection is…

3. Understanding the preventive measures for HPV is…

4. Understanding HPV screening methods is…

5. Understanding the impacts of various HPV vaccine types (valence) on preventing disease in different sexes is…

6. Understanding the health benefits of HPV vaccination is…

7. Understanding the health benefits of HPV vaccination in different sexes is…

8. Understanding HPV prevention information provided by healthcare professionals is…

9. Identifying the accuracy of information on HPV is…

10. Determining the completeness of HPV prevention information is…

11. Evaluating whether HPV infection prevention information applies to me is…

12. Determining the consistency of the HPV information obtained is…

13. Determining the credibility of the HPV information obtained is…

14. Determining if I need the HPV vaccine is…

15. Discerning which type (valence) of HPV vaccine is suitable for me based on the obtained information on HPV prevention is…

16. Using HPV-related information to help me choose the appropriate HPV type (valence) vaccine is…

Factor 2: Seeking information from healthcare professionals

17. Asking healthcare professionals about prevention measures for HPV infection is…

18. Expressing a request for HPV screening with healthcare professionals is…

19. Asking healthcare professionals about treatments for HPV-related diseases is…

20. Confirming the accuracy of my understanding of HPV infection prevention information with healthcare professionals is…

21. Discussing HPV issues with healthcare professionals is…
